# Higher-Order Models for Resonant Viscosity and Mass-Density Sensors

**DOI:** 10.3390/s20154279

**Published:** 2020-07-31

**Authors:** Thomas Voglhuber-Brunnmaier, Bernhard Jakoby

**Affiliations:** Institute for Microelectronics and Microsensors, Johannes Kepler University, 4040 Linz, Austria; bernhard.jakoby@jku.at

**Keywords:** fluid model, hydrodynamic function, fluid sensor, Reynolds number

## Abstract

Advanced fluid models relating viscosity and density to resonance frequency and quality factor of vibrating structures immersed in fluids are presented. The numerous established models which are ultimately all based on the same approximation are refined, such that the measurement range for viscosity can be extended. Based on the simple case of a vibrating cylinder and dimensional analysis, general models for arbitrary order of approximation are derived. Furthermore, methods for model parameter calibration and the inversion of the models to determine viscosity and/or density from measured resonance parameters are shown. One of the two presented fluid models is a viscosity-only model, where the parameters of it can be calibrated without knowledge of the fluid density. The models are demonstrated for a tuning fork-based commercial instrument, where maximum deviations between measured and reference viscosities of approximately ±0.5% in the viscosity range from 1.3 to 243 mPas could be achieved. It is demonstrated that these results show a clear improvement over the existing models.

## 1. Introduction

When electromechanical transducers are immersed in a fluid, the mechanical properties of the fluid influence the resonance spectrum measured at the electrical terminals [1]. Based on the theory of Sauerbrey [2], quartz thickness shear mode resonators have long been used to measure smallest mass depositions on these sensors, e.g., in vapor deposition chambers under close to vacuum conditions. An equivalent to the Sauerbrey equation for fluids has been developed by Kanazawa and Gordon [3] and found wide application in measurement practice following the work of Martin [4]. In the subsequent years, the sensitivity of acoustic fluid sensors was enhanced by replacing these bulk acoustic wave devices by surface acoustic wave sensors [5]. The drawback of using such sensors featuring dominant shearing motion, is that that according to the one-dimensional model, only the density-viscosity product can be measured, but not the individual parameters. The ability to separate density ρ and viscosity η due to spurious effects (e.g., finite area of vibration and boundary effects) is very limited for shear sensors and yields large measurement errors on the individual parameters [6]. Although this problem could be solved by adding liquid traps (see e.g., [7,8]), such sensors are not widely used in measurement practice, likely due to demanding cleaning requirements.

Piezoelectric cantilevers [9,10,11,12,13] and quartz tuning fork sensors [14,15,16,17] are excellent candidates for simultaneous determination of density and viscosity, when the resonance characteristics of the immersed sensors are evaluated. Contrary to shear wave sensors, where the relationship between the resonance parameters and ρ, η is simple, the problem is profound e.g., for vibrating cantilevers in general. Even when the cantilever is assumed with infinite length, such that the problem can be reduced to two dimensions, closed form solutions are only available for cylindrical cross-sections [18,19]. A numerically determined correction factor, boundary element methods, and tabulated values for the hydrodynamic loading for rectangular cross-sections are found in [11,20,21], respectively. However, the numerical modeling approach for vibrating rectangular cross-sections [21] is already involved, and closed form expressions are only available in some limiting cases, such as for zero viscosity or for infinitely extended plates.To our best knowledge, finding closed form solutions for arbitrary geometries is a hopeless endeavor, but the significance of such forms is questionable in measurement practice, in particular since fabrication and material tolerances are encountered. In order to achieve measurement accuracies which are on par with lab grade instruments, additional model parameter adjustments using test liquids are always required. It is therefore more suitable to work with parametrized models which approximate the physical problem well and can be adjusted by calibration measurements. In the derivation of such parametrized models, the hydrodynamic function [11], which essentially describes the fluid resistance acting against a vibrating body, is central. This fluid force constitutes a boundary condition for the resonator changing its resonance frequency and *Q*-factor. It is therefore possible to establish a relationship between the physical fluid properties and the measured resonance parameters. The task of the sensor designer is to guarantee that the resonance parameters can be correctly measured and that a fluid model is available which can be transformed to yield unique and accurate fluid parameters from the resonance parameters. As the exact hydrodynamic function is only known for some special cases, the conventionally used fluid models derived, e.g., by [22,23,24,25,26] are all based on an approximation of the hydrodynamic function of a vibrating prismatic beam featuring a cylindrical cross-section by means of a truncated power series. A remarkable fact, shown by a many researchers in experimental work [22,23,24,25,26,27,28,29,30,31], is that the resulting reduced order models with adjustable parameters are suitable for representing various different types of fluid loaded vibrating structures, including cantilevers of different cross-sections, large steel and miniaturized quartz tuning forks, spiral springs, platelets, U-shaped wires, piezo buzzers in fluids, etc. However, the limitations of the approximation being made to obtain models of manageable complexity, narrows the accurately measurable range of fluid parameters. The unified fluid models presented in this work, follow in a straightforward manner from the work of [11,22,27]. It encompasses the previous ones but can be extended to arbitrary order of approximation. The key features of these models are that any order of approximation can be achieved, given enough calibration measurements. Furthermore, the complexity of the fluid parameter determination from resonance parameters remains manageable for any order. As a side effect, also a viscosity-only model is derived which needs no information about the fluid density for calibrating the model parameters.

The remainder of the paper is structured as follows: In Section 2, the theoretical background is established. The two new arbitrary order fluid models in Equations (16) and (24) are derived in Section 2.4 and Section 2.5. Numerical experiments for the vibrating cylinder and analyzes of measured data obtained using a piezoelectric tuning fork sensor are shown in Section 3. Two fundamental, but longer treatises on dimensional analysis and resonance parameters are found in the Appendixes Appendix A and Appendix B.

## 2. Materials and Methods

The closed form solution for the vibrating cylinder is chosen as a vehicle to reveal functional dependencies which apply also for more complex cross-sections, but for which no closed forms are known.

### 2.1. Vibrating Cylinder Immersed in Viscous Fluid

The complex valued fluid force amplitude per length ${\underline{F}}^{\prime }$ acting against the oscillatory motion of an infinitely long prismatic cylinder is given by [19,23]

(1) $$\begin{array}{cc} \; & \underline{F^{\prime }}=j\omega \underline{v}m^{\prime }\underline{\Gamma }\left(\beta \right),\\ \; & \mathrm{with} \end{array}$$

(2) $$\begin{array}{cc} \; & \underline{\Gamma }\left(\beta \right)=1+\frac{4K_{1}\left(\sqrt{j\beta }\right)}{\sqrt{j\beta }K_{0}\left(\sqrt{j\beta }\right)}, \end{array}$$

(3) $$\begin{array}{cc} \; & m^{\prime }=\rho R^{2}\pi , \end{array}$$

(4) $$\begin{array}{cc} \; & \beta =R^{2}\omega \rho /\eta . \end{array}$$

The fluid is considered incompressible and Newtonian. The radius of the cylinder, the velocity amplitude of the rigid cross-section, and the angular frequency are denoted by *R*, *v*, and ω, respectively. The relation between time domain and complex amplitude is given, e.g., for the vibration velocity by $v(t)=R\{\underline{v}\exp(j\omega t)\}$. K${}_{0}$ and K${}_{1}$ denote the modified Bessel functions of second kind and *j* is the imaginary unit $\sqrt{-1}$. The function $\underline{\Gamma }$ assumes the value of 1 for vanishing viscosity (i.e., β→∞). The associated flow field in this case is that of the potential flow [32], where the mass/length $m^{\prime }$ is periodically displaced, but no viscous losses occur. For non-zero viscosity, the real part of $\underline{\Gamma }$ encompasses how viscous effects add additional mass drag and the imaginary part represents viscous losses. The function is therefore also termed *hydrodynamic function*. We demand that the vibration amplitudes are small enough that the relation between force and velocity amplitudes remain in the linear range. This means that the convective part in the Navier–Stokes equations causing non-linearity and fluid instability is neglected. Although not generally permissible, this assumption holds for many vibrators, especially for QCMs [33], QTFs and micro cantilevers [27]. It is then convenient to eliminate the driving velocity and to consider the fluid impedance per unit length

(5) $$\underline{Z^{\prime }}=\underline{F^{\prime }}/\underline{v}=j\omega m^{\prime }\underline{\Gamma }\left(\beta \right),$$

henceforth. It is essential to note that $\underline{\Gamma }$ and β are both dimensionless variables, where the latter is termed *Reynolds number* [11], or *non-dimensional frequency* [18]. Furthermore, it is a coincidence that the equivalent moved mass per length $m^{\prime }$ equals the area of the cylinder cross-section times the fluid density. This is generally not the case for other geometries. The derivation of Equation (2) for this very basic geometry is already involved with details shown, for example, in [19]. The determination of the fluid parameters from the fluid loaded resonator is difficult, as ρ and η appear also in the arguments of two Bessel functions. The fluid models presented in this work, are based on an expansion the hydrodynamic function in Equation (2) into a power series around β→∞, yielding

(6) $$\begin{array}{cc} \underline{\Gamma } & =1+\frac{4}{{\left(j\beta \right)}^{1/2}}+\frac{2}{j\beta }-\frac{1}{2{\left(j\beta \right)}^{3/2}}+\frac{1}{2{\left(j\beta \right)}^{2}}-\frac{25}{32{\left(j\beta \right)}^{5/2}}+\frac{13}{8{\left(j\beta \right)}^{3}}+H.O.T, \end{array}$$

(7) $$\begin{array}{cc} \underline{\Gamma } & \approx 1+\frac{4}{\sqrt{2\beta }}-j\left(\frac{4}{\sqrt{2\beta }}+\frac{2}{\beta }\right). \end{array}$$

For the reduced order models [22,23,24,25,26], the approximation of the fluid force in Equation (7) is added to the equation of motion of the resonator (see Section 2.3), yielding models for the resonance parameters of the fluid loaded resonator shown in Equation (12). A consequence of the agreement between Equation (7) and Equation (2) being best for large β is that the model approximation is more accurate for thicker cylinders vibrating at higher frequencies. By introducing the characteristic decay length of plane shear waves δ

(8) $$\beta =\frac{\rho R^{2}\omega }{\eta }=2{\left(\frac{R}{\delta }\right)}^{2}\;\;\;\mathrm{with}\;\;\;\delta =\sqrt{\frac{2\eta }{\rho \omega }},$$

it also becomes clear that the decay length should be small compared to the dimension of the vibrating cylinder. Consequently, the reduced fluid models are only applicable up to a certain maximum viscosity for a given resonator.

### 2.2. Arbitrary Cross-Sections Vibrating in Fluid

**Figure 1:**
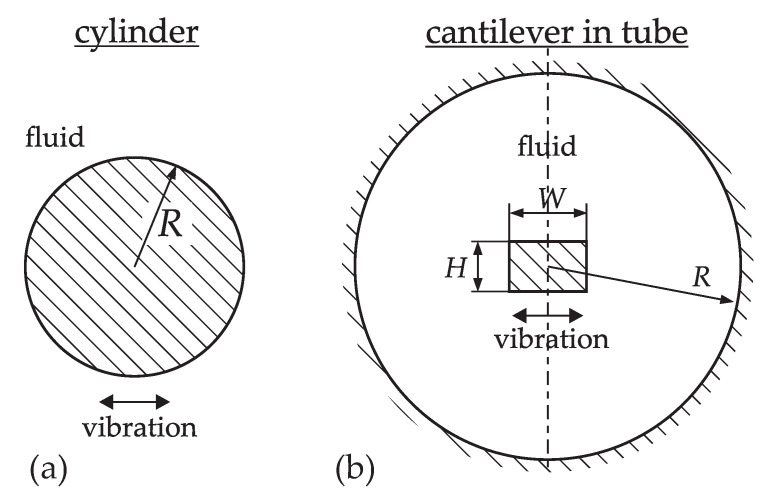
Prismatic beam with cylindrical cross-section (**a**) and a cantilever vibrating in a tube (**b**) as examples for a simple and a complex geometry of a vibrating structure.

In general, hydrodynamic functions for different cross-sections must be determined numerically. For the numerical modeling of complex geometries the characteristic variables are not easily recognized in the modeling approach. By means of a dimensional analysis, with details given in the Appendix A, however, it can be shown that the dependence of the hydrodynamic function on the fluid properties is in all cases determined exclusively by a single Reynolds number β. The additional characteristic parameters required to describe the problem completely can be expressed by additional aspect ratios $\alpha_{i}$ as will be illustrated in the following. For the simple cylinder versus a rectangular cantilever vibrating in a tube (see Figure 1), for instance, follows

(9) $$\begin{array}{cccc} \; & \underline{\mathrm{cylinder}}: & \; & \underline{\mathrm{cantilever}\;\mathrm{in}\;\mathrm{tube}:}\\ \; & \underline{Z^{\prime }}=j\omega A\rho \;\underline{\Gamma }\left(\beta \right), & \underline{Z^{\prime }} & =j\omega A\rho \;\underline{\Gamma }(\beta ,\alpha_{1},\alpha_{2}),\\ \; & A=R^{2}\pi , & A & =W\;H,\\ \; & \beta =R^{2}\omega \rho /\eta , & \beta & =\max{\left(W,H\right)}^{2}\omega \rho /\eta ,\\ \; & \; & \alpha_{1} & =W/H,\;\;\alpha_{2}=W/R. \end{array}$$

As the additional aspect ratios $\alpha_{1}$ and $\alpha_{2}$ are fixed for a given sensor geometry, the hydrodynamic function can be considered to be depending only on β for any cross-section and configuration. The method to derive a reduced order model from the simple cylindrical geometry can therefore be generally applied, but it must be kept in mind that the shape of the hydrodynamic functions is unknown and may be more complex, requiring higher orders of approximation. An indicator for requiring higher orders is, if any involved dimension is comparably small to the decay length δ given in Equation (8).

### 2.3. Resonator Model

The fluid forces $\underline{F^{\prime }}$ represent a boundary condition for the immersed resonator changing its resonance parameters. These resonance parameters are defined as characteristics of the mechanical admittance spectrum and the dependence of these parameters on the hydrodynamic fluid loading is outlined in the following.

In the vicinity of a resonance, a vibrating structure can be approximated by an equivalent spring-mass-damper system with the respective parameters $\bar{k}$, $\bar{m}$, and $\bar{c}$ and the additional fluid mass loading $m_{f}$ and damping $c_{f}$. The acoustic admittances $\underline{Y}=\underline{v}/\underline{F}$ of such a system is given by

(10) $$\underline{Y}\left(\omega \right)=\frac{1}{j\omega \left(\bar{m}+m_{f}\right)+\left(\bar{c}+c_{f}\right)-j\bar{k}/\omega }.$$

**Figure 2:**
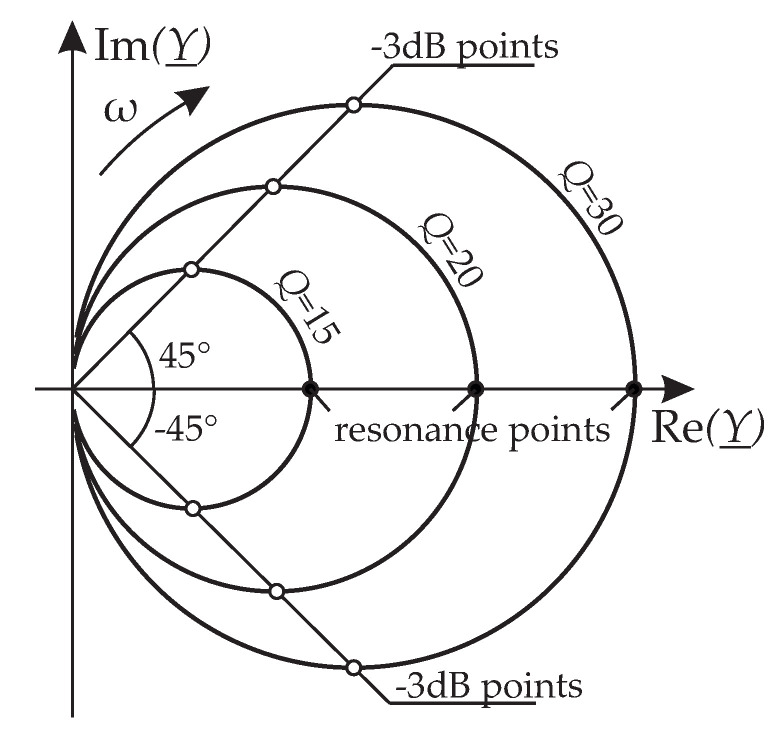
Nyquist plots of the mechanical admittance of three resonances.

The Nyquist plots of such admittances resemble circles as is shown in Figure 2. The resonance frequency is defined at the point where the function is real and maximum. The bandwidth Δω used to calculate the *Q*-factor by $Q=\omega_{r}/\Delta \omega$ lies between the $\pm 45^{\circ }$ phase angle points. For driven resonances, it is therefore favorable to use the admittance *Y*. (Contrary to when force-displacement relations of driven resonators, or free vibrations, e.g., measured in ring-down mode, are considered.) The resonance parameters are given by

(11) $$\begin{array}{c} \omega_{r}=\sqrt{\frac{\bar{k}}{\bar{m}+m_{f}}}\;\;\;\mathrm{and}\;\;\;Q_{r}=\frac{\bar{k}}{\omega_{r}}\frac{1}{\bar{c}+c_{f}}. \end{array}$$

A relation between resonance parameters and fluid parameters can be obtained by splitting Equation (7) in real and imaginary part and attributing them to added fluid mass $m_{f}$ and damping $c_{f}$ (see e.g., Appendix B for details). Some of the established models, which are all based on similar derivations and that can in principle be converted into each other (In case of free instead of forced oscillations, expressions for the resonance parameters have to be adapted.) are:

(12) $$\begin{array}{ccccc} \; & \mathrm{Heinisch}\;\mathrm{et}\;\mathrm{al}.: & \; & \; & \mathrm{Youssry}\;\mathrm{et}\;\mathrm{al}.:\\ \omega_{r}= & {\left(m_{0k}+m_{\rho k}\rho +m_{\eta \rho k}\sqrt{\frac{\eta \rho }{\omega_{r}}}\right)}^{-1/2} & \; & \;\;\;\;\;\;g_{2}= & M_{L}\left(a_{1}+a_{2}\sqrt{\frac{2\eta }{\rho \omega_{r}b^{2}}}\right)\\ Q_{r}= & \frac{1}{\omega_{r}}{\left(c_{0k}+c_{\eta k}\eta +c_{\eta \rho k}\sqrt{\rho \eta \omega_{r}}\right)}^{-1} & \; & \;\;\;\;\;\;g_{1}= & M_{L}\omega_{r}\left(b_{1}\sqrt{\frac{2\eta }{\rho \omega_{r}b^{2}}}+b_{2}\frac{2\eta }{\rho \omega_{r}b^{2}}\right)\\ \; & \; & \;\\ \; & \mathrm{Toledo}\;\mathrm{et}\;\mathrm{al}.,\;\mathrm{Dufour}\;\mathrm{et}\;\mathrm{al}.:\; & \; & \; & \mathrm{Zhang}\;\mathrm{et}\;\mathrm{al}.:\\ g_{1}= & C_{1}\sqrt{f_{r}}\sqrt{\rho \eta }+C_{2}\eta & \; & \;\;\frac{1}{Q_{r}}\frac{\omega_{0}^{2}}{\omega_{r}^{2}}= & A\sqrt{\frac{\rho \eta }{\omega_{r}}}+B\frac{\eta }{\omega_{r}}\\ g_{2}= & C_{3}\rho +\frac{C_{4}}{\sqrt{f_{r}}}\sqrt{\rho \eta } & \; & \frac{\omega_{0}^{2}}{\omega_{r}^{2}}-1= & A\sqrt{\frac{\rho \eta }{\omega_{r}}}+C\rho \end{array}$$

Here, $Q_{r}$, $\omega_{r}=2\pi f_{r}$ and $\omega_{0}$ denote *Q*-factor and angular resonance frequency in the fluid and in vacuum, respectively. $g_{1}$ and $g_{2}$ are functions of the resonance parameters which correspond to the real and imaginary parts of the hydrodynamic function. It is apparent that the number of parameters of the models $m_{0k},m_{\rho k},m_{\eta \rho k},c_{0k},c_{\eta k},c_{\eta \rho k}$ for [23], $C_{1},C_{2},C_{3},C_{4}$ for [22,25], $M_{L},a_{1},a_{2},b_{1},b_{2}$ for [24] or A,B,C for [26] differ. For instance, the model from Heinisch et al [23] where the vacuum resonance parameters $\omega_{0}$ and $Q_{0}$ are fitted in the calibration procedure ($m_{0k},c_{0k}$), are considered known in the model of Zhang et al. [26]. Furthermore, $c_{\eta \rho k}$ and $m_{\eta \rho k}$ are allowed to differ, while according to Equation (7), the same $1/\sqrt{\beta }$ dependency in real and imaginary part of the hydrodynamic function suggests that $c_{\eta \rho k}$ and $m_{\eta \rho k}$ should be equal as in the model of Zhang et al. [26] (parameter *A*). Consequently, the model from Heinisch et al., yields lower deviations between the fluid reference values and the measured values, as is also outlined in [26]. For the above mentioned models, the closed form solutions for the fluid force of vibrating cylinders have been approximated by a power series in the Reynolds number, where a low order of approximation was chosen in order to obtain models which can be rearranged yielding simple expressions for η and ρ [29].

### 2.4. Higher-Order Fluid Models

The extension to higher-order approximation is motivated by using the expression for the resonance parameters derived in Appendix B, where $\rho_{s}$ represents the resonator mass-density

(13) $$\begin{array}{c} \omega_{r}=\frac{\omega_{0}}{\sqrt{1+\frac{\rho }{\rho_{s}}\Gamma_{R}}},\;\;\;\mathrm{and}\;\;\;Q_{r}=\frac{\omega_{0}}{\omega_{r}}{\left(\frac{1}{Q_{0}}+\frac{\omega_{r}}{\omega_{0}}\frac{\rho }{\rho_{s}}\Gamma_{I}\right)}^{-1}. \end{array}$$

These relations can also be found in [11], for instance. However, in this work, alternative scales are used which render the equations more convenient for our approach. $\Gamma_{R}$ and $\Gamma_{I}$ represent the real and the negative imaginary parts of the hydrodynamic function i.e., $\underline{\Gamma }=\Gamma_{R}-j\Gamma_{I}$. Following the usual derivation of the fluid model for the cylindrical cross-section, but without the early truncation of Equation (6), yields

(14) $$\begin{array}{cc} \Gamma_{R} & =\frac{\omega_{0}^{2}}{\omega_{r}^{2}}-1=\frac{\rho }{\rho_{s}}\left(1+\frac{4}{{\left(2\beta \right)}^{1/2}}+\frac{1}{{\left(2\beta \right)}^{3/2}}-\frac{2}{{\left(2\beta \right)}^{4/2}}+\frac{25}{8{\left(2\beta \right)}^{5/2}}+H.O.T.\right), \end{array}$$

(15) $$\begin{array}{cc} \Gamma_{I} & =\frac{\omega_{0}^{2}}{\omega_{r}^{2}}\frac{1}{Q_{r}}-\frac{\omega_{0}}{\omega_{r}}\frac{1}{Q_{0}}=\frac{\rho }{\rho_{s}}\left(\frac{4}{{\left(2\beta \right)}^{1/2}}+\frac{4}{{\left(2\beta \right)}^{2/2}}-\frac{1}{{\left(2\beta \right)}^{3/2}}+\frac{25}{8{\left(2\beta \right)}^{5/2}}-\frac{13}{{\left(2\beta \right)}^{6/2}}+H.O.T.\right). \end{array}$$

From Equation (8) follows the proportionality $1/\sqrt{\beta }\propto \sqrt{\nu /\omega_{r}}$, with ν denoting the kinematic viscosity ν=η/ρ. It can be expected that for vibrating cross-sections of alternative shape, the constants of the polynomial are different. By introducing the new variable $\xi =\sqrt{\nu /\omega_{r}}$, attributing $\rho_{s}$ to the constants ($a_{i}$ and $b_{i}$) and neglecting higher orders than $N_{a}$ and $N_{b}$, the general model follows

(16) $$g_{a}=\rho \left(a_{0}+a_{1}\xi +\cdots +a_{N_{a}}\xi^{N_{a}}\right)\;\;\;\mathrm{and}\;\;\;g_{b}=\rho \left(b_{1}\xi +\cdots +b_{N_{b}}\xi^{N_{b}}\right).$$

Although $g_{a}$ and $g_{b}$ are equal to $\Gamma_{R}$ and $\Gamma_{I}$, i.e.,

(17) $$\begin{array}{c} g_{a}=\frac{\omega_{0}^{2}}{\omega_{r}^{2}}-1\;,\;\;\mathrm{and}\;\;\;g_{b}=\frac{\omega_{0}^{2}}{\omega_{r}^{2}}\frac{1}{Q_{r}}-\frac{\omega_{0}}{\omega_{r}}\frac{1}{Q_{0}}, \end{array}$$

a distinction is made, to emphasize that $g_{a}$ and $g_{b}$ are functions of the resonance parameters that are measured. It is assumed that the constants $a_{i}$ and $b_{i}$ are known from a model calibration procedure and $g_{a}$ and $g_{b}$ are calculated from the resonance parameters provided by a resonance estimation algorithm. ξ and density ρ are therefore the two unknowns to be determined. The density ρ can be eliminated from Equation (16) yielding a polynomial in ξ alone

(18) $$\left(g_{b}a_{0}-g_{a}b_{0}\right)+\left(g_{b}a_{1}-g_{a}b_{1}\right)\xi +\cdots +\left(g_{b}a_{N}-g_{a}b_{N}\right)\xi^{N}=0,$$

where $N=\max(N_{a},N_{b})$. In case of $N_{b}<N_{a}$ follows that $b_{N_{b}\cdots N_{a}}=0$ or $a_{N_{b}\cdots N_{a}}=0$ for $N_{b}>N_{a}$. Additionally, $Q_{r}=Q_{0}$ at ξ=0, and therefore $b_{0}=0$. The constants of the polynomial are all known such that an estimate for the kinematic viscosity is obtained by $\hat{\nu }=\xi^{*2}\omega_{r}$, where $\xi^{*}$ denotes the correct real root of Equation (18). Subsequently, an estimate of density $\hat{\rho }$ is obtained by substituting $\xi^{*}$ in Equation (16) and dynamic viscosity follows from $\hat{\eta }=\hat{\nu }\hat{\rho }$.

A comparison with the models in Equation (12) shows that they all can be reduced to

(19) $$\begin{array}{c} g_{a}=\rho \left(a_{0}+a_{1}\xi \right)\;\;\;\mathrm{and}\;\;\;g_{b}=\rho \left(b_{1}\xi +b_{2}\xi^{2}\right), \end{array}$$

which represents a special case ([0,1][1,2]) of the extended model. For equivalence with the model of Heinisch et al. and Zhang et al., for instance, the parameters are related by

(20) $$m_{0k}=\omega_{0}^{-2},\;m_{\rho k}=a_{1}\omega_{0}^{2},\;m_{\eta \rho k}=a_{2}\omega_{0}^{2},\;c_{0k}={\left(\omega_{0}Q_{0}\right)}^{-1},\;c_{\eta k}=b_{2}\omega_{0}^{2},\;c_{\eta \rho k}=b_{1}\omega_{0}^{2}$$

or

(21) $$A=a_{1},A=b_{1},B=b_{2},C=a_{0},Q_{0}\to \infty ,$$

respectively.

#### 2.4.1. Model Calibration

Given *M* calibration measurements, Equation (16) can be transformed into matrix form

(22) $$\underset{g_{a}}{\underset{︸}{\left[\begin{array}{c} g_{a,1}\\ \vdots \\ g_{a,M} \end{array}\right]}}=\underset{\Xi_{a}}{\underset{︸}{\left[\begin{array}{cccc} \rho_{1} & \rho_{1}\xi_{1} & \cdots & \rho_{1}\xi_{1}^{Na}\\ \vdots & \vdots & \ddots & \vdots \\ \rho_{M} & \rho_{M}\xi_{M} & \cdots & \rho_{M}\xi_{M}^{Na} \end{array}\right]}}\cdot \underset{a}{\underset{︸}{\left[\begin{array}{c} a_{0}\\ \vdots \\ a_{Na} \end{array}\right]}}\;\;\mathrm{and}\;\;\underset{g_{b}}{\underset{︸}{\left[\begin{array}{c} g_{b,1}\\ \vdots \\ g_{b,M} \end{array}\right]}}=\underset{\Xi_{b}}{\underset{︸}{\left[\begin{array}{ccc} \rho_{1}\xi_{1} & \cdots & \rho_{1}\xi_{1}^{Nb}\\ \vdots & \ddots & \vdots \\ \rho_{M}\xi_{M} & \cdots & \rho_{M}\xi_{M}^{Nb} \end{array}\right]}}\cdot \underset{b}{\underset{︸}{\left[\begin{array}{c} b_{1}\\ \vdots \\ b_{Nb} \end{array}\right]}}.$$

The ranges of viscosity and density of the test fluids define the valid calibration range. A least square fit for the parameter vector a and b can be obtained using the Moore–Penrose inverse (where W is an identity matrix)

(23) $$\begin{array}{cc} \; & \hat{a}={\Xi_{a}}^{†}\cdot g_{a}\;\;\mathrm{and}\;\;\hat{b}={\Xi_{b}}^{†}\cdot g_{b}\;\;\;\;\mathrm{with}\;\;\;\;\Xi^{†}={\left(\Xi^{T}\cdot W\cdot \Xi \right)}^{-1}\cdot \Xi^{T}\cdot W. \end{array}$$

However, the least squares optimization favors larger relative deviations in the lower viscosity range, so that W should be adjusted in order to achieve the wanted distribution of the unavoidable model deviations for $M>N_{a}+1$ and $M>N_{b}$. With a sufficiently high number of calibration measurements available, the order of the approximation can be expanded as desired.

### 2.5. Viscosity-Only Model

For the calibration of the previous model, the densities and viscosities of various test liquids where required. However, it is possible to perform model calibration and measurement of the kinematic viscosity without knowledge of fluid density. Rearranging Equation (13) for the fluid loss factor $g=\Gamma_{I}/\Gamma_{R}$ yields a model which depends only on the kinematic viscosity ν but not on density ρ

(24) $$g\left(\xi \right)=\frac{g_{b}}{g_{a}}\left(\xi \right)=\frac{b_{1}\xi +\cdots +b_{N_{b}}\xi^{N_{b}}}{a_{0}+a_{1}\xi +\cdots +a_{N_{a}}\xi^{N_{a}}}\approx c_{1}\xi +\cdots +c_{N_{c}}\xi^{N_{c}}.$$

The rational function of the fluid loss factor can itself be approximated by a polynomial in ξ. The unknown ξ can be calculated for given constants $c_{i}$ and a measurement point *g* by finding the correct root $\xi^{*}$ of Equation (24). Subsequently, the kinematic viscosity results with $\hat{\nu }=\xi^{*2}\omega_{r}$. The dynamic viscosity η, however, cannot be determined using this particular model.

#### Model Parameter Calibration

A procedure for model calibration similar to that in Section 2.4.1 can be established

(25) $$\underset{g}{\underset{︸}{\left[\begin{array}{c} g_{1}\\ \vdots \\ g_{M} \end{array}\right]}}=\underset{\Xi_{c}}{\underset{︸}{\left[\begin{array}{cccc} 1 & \xi_{1} & \cdots & \xi_{1}^{Nc}\\ \vdots & \vdots & \ddots & \vdots \\ 1 & \xi_{M} & \cdots & \xi_{M}^{Nc} \end{array}\right]}}\cdot \underset{c}{\underset{︸}{\left[\begin{array}{c} c_{1}\\ \vdots \\ c_{Nc} \end{array}\right]}}.$$

The constants are again obtained by a weighted least squares fit

(26) $$\hat{c}={\Xi_{c}}^{†}\cdot g_{c}.$$

It is noteworthy that although the resonance parameters depend on both density and viscosity, a fluid model can be found which depends solely on the kinematic viscosity ν. The question of whether dynamic (η) or kinematic viscosity should be regarded as the primary viscosity parameter can be decided on the basis of this model in favor for the kinematic viscosity ν.

In this section, it was shown that extending the usual fluid models to higher polynomial order is straightforward. Given Equations (13) and (A17), it is apparent that also the extended models rely on power series approximations of the real and imaginary parts of the hydrodynamic function. Although polynomials in ξ were used for the presented models, it should be kept in mind that any function suitably approximating the hydrodynamic function could be used instead.

## 3. Results and Discussion

Synthetic data, generated using the closed form solution for the cylinder, as well as experimental data obtained with quartz tuning fork sensors, are analyzed. For the latter, the hydrodynamic function is estimated from measured data and compared to theoretical predictions. The deviations between the reference values of certified standard fluids and estimates using different model orders, are analyzed in detail. It will be shown that with increasing order, the systematic model deviations reduce to a point where measurement errors and presumably deviations between actual and nominal fluid parameters or unnoticed fluid degradations become dominant.

### 3.1. Numerical Analysis for the Vibrating Cylinder

**Figure 3:**
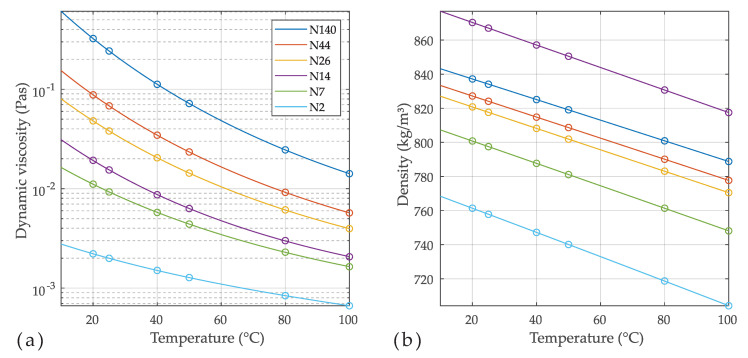
Dynamic viscosities (**a**) and densities (**b**) of various NIST-traceable viscosity reference fluids (${}^{\circ }$). The lines represent temperature models [34]. The numbers of the standards coarsely agree with the kinematic viscosity in cSt at 40 ${}^{\circ }$C, i.e., ν of N14 is approx. 14 cSt at 40 ${}^{\circ }$C.

**Figure 4:**
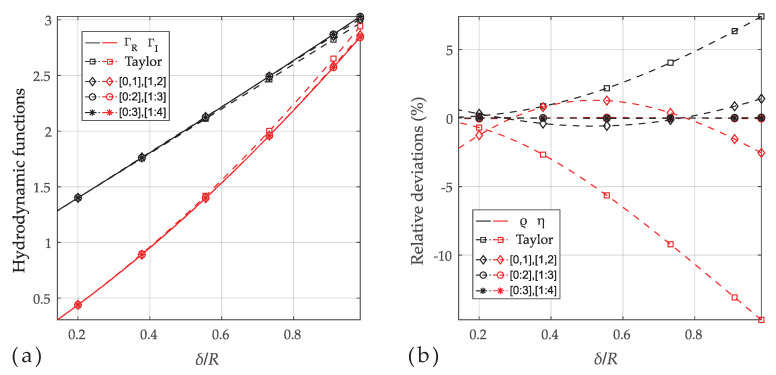
(**a**) Real (black) and imaginary part (red) of the hydrodynamic function (solid line) in Equation (2), power series model in Equation (7) (□) and three different orders of approximations (e.g., [0:2],[1:2] means that coefficients $a_{0}\cdots a_{2}$ and $b_{1}\cdots b_{3}$ in Equation (16) are non-zero). (**b**) The deviations between reference and estimated viscosities and densities. Deviations are reduced with increasing order and remain below 0.015% for [0:3],[1:4] (*).

Equations (2) and (13) were used to generate resonance parameters $\omega_{r}$ and $Q_{r}$ for a resonator with vacuum resonance frequency 32.768 kHz (i.e., $\omega_{0}=205.89\cdot 10^{3}\;$s${}^{-1}$) and unloaded *Q*-factor of $Q_{0}=10^{4}$. The fluid properties correspond to that of a N140 fluid standard at various temperatures, as shown in Figure 3. The resonator radius and density are defined with R=0.1 mm and $\rho_{s}=2800\;$kg/m${}^{3}$, respectively. The correct hydrodynamic function expression from Equation (2) (solid line in the left Figure 4) was used to generate the resonance parameters $\omega_{r}$ and $Q_{r}$ for the fluid parameters of N140 in the temperature range from 10 ${}^{\circ }$C to 100 ${}^{\circ }$C. The dashed lines in Figure 4a represent the power series approximation of different order in Equations (14) and (15) (□,⋄,∘,∗). In (b), the deviations between reference and calculated viscosities and densities are shown. It is apparent that the first order power series model is accurate only around β→∞ which corresponds to δ/R→0 (see Equation (8)). The approximation (⋄) [0,1],[1,2] is of the same order as the power series model (□), but the parameters $a_{0},a_{1},b_{1}$ and $b_{2}$ were adjusted such that the relative deviations are minimized on average, yielding deviations which are approximately 5 times smaller at the higher viscosities. The deviations due to model approximation can be made arbitrarily small by increasing the order of the approximation, which is a confirmation for the validity of the approach.

### 3.2. Quartz Tuning Fork Measurements

The measurement results were obtained with the VDC100 measurement cell and the MFA200 resonance analyzer, both provided by MicroResonant [35]. Details on the instrument and the dimensions of the QTF can be found e.g., in [36,37]. The parameters of the reference fluids and the measured resonance parameters are shown in Table 1. The standard fluids were measured at different temperatures to enhance the use of the fluids.

#### 3.2.1. Extended Viscosity and Density Model

The set of four fluids # 2, 13, 19, and 23 in Table 1 were used for calibrating the parameters $a_{0}\cdots a_{3}$ and $b_{1}\cdots b_{4}$ of the model

(27) $$\begin{array}{c} g_{a}=\rho \left(a_{0}+a_{1}\bar{\xi }+a_{2}{\bar{\xi }}^{2}+a_{3}{\bar{\xi }}^{3}\right)\;\;\;\mathrm{and}\;\;\;g_{b}=\rho \left(b_{1}\bar{\xi }+b_{2}{\bar{\xi }}^{2}+b_{3}{\bar{\xi }}^{3}+b_{4}{\bar{\xi }}^{4}\right). \end{array}$$

Scaling $\bar{\xi }=\xi /\max\left(\xi \right)$ was introduced so that the constants have the same physical units and the orders of magnitude are more similar. Using the estimated vacuum resonance parameters $\omega_{0}=205.818\;$s${}^{-1}$ and $Q_{0}=14100$, the determined constants are (in m${}^{3}$/kg)

(28) $$\begin{array}{cccc} a_{0}=2.9983\times 10^{-4}, & a_{1}=2.2803\times 10^{-4}, & a_{2}=5.1036\times 10^{-6}, & a_{3}=\;\;\;6.0255\times 10^{-8},\\ b_{1}=2.3219\times 10^{-4}, & b_{2}=1.4708\times 10^{-5}, & b_{3}=6.7354\times 10^{-5}, & b_{4}=-3.0329\times 10^{-5}. \end{array}$$

**Figure 5:**
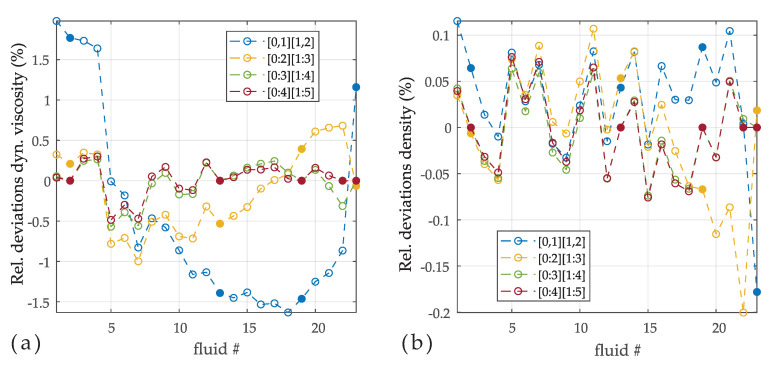
(**a**) Relative deviations of dynamic viscosity η for different polynomial orders. The filled dots were used for calibrating the model constants. Fluid #22 was added for calibrating the highest order [0:4][1:5]. (**b**) Relative deviations for density ρ. Although for viscosity relative deviations were reduced using higher orders, the effect on density is less pronounced.

**Figure 6:**
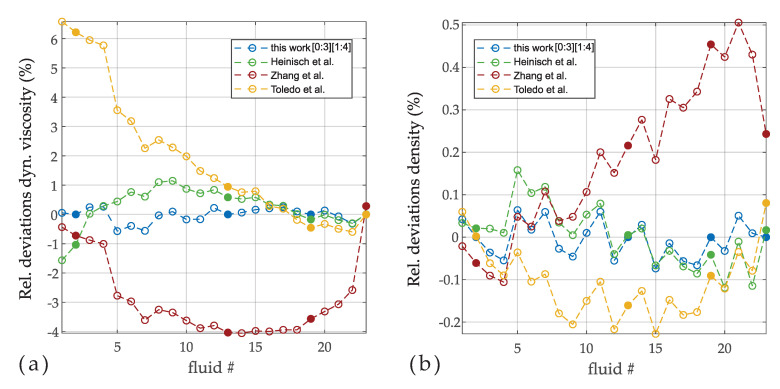
(**a**) Relative deviations of dynamic viscosity η for different models. The filled dots were used for calibrating the model constants. (**b**) Relative deviations for density ρ.

Figure 5 shows the deviations between reference and measured values of dynamic viscosity and density for different orders of the polynomials. The order which corresponds to the models in Equation (12) is [0,1] and [1,2] (i.e., $a_{0},a_{1},b_{1},b_{2}$) showing deviations in the range of ±1.5%.Order extension reduces the deviations gradually, such that deviations of −0.57% to 0.22% are achieved over the full viscosity range from 1.27 mPas to 242.9 mPas using the parameters in Equation (28). Adding a further point to the calibration (#22) and increasing the orders by one does not enhance the agreement significantly. Interestingly, the deviations on density reduce only slightly with increasing order. In Figure 6 the various models in Equation (12) are compared with an extended model presented in this work. The models were calibrated using the same fluid as before with no weighting (i.e., W is an identity matrix, see Equation (23)) applied. The deviations are differently distributed for the various models, but which is mostly coincidentally. Although all models yield reasonable results, it apparent that the models using more parameters are superior for minimizing deviations.

#### 3.2.2. Viscosity-Only Model

**Figure 7:**
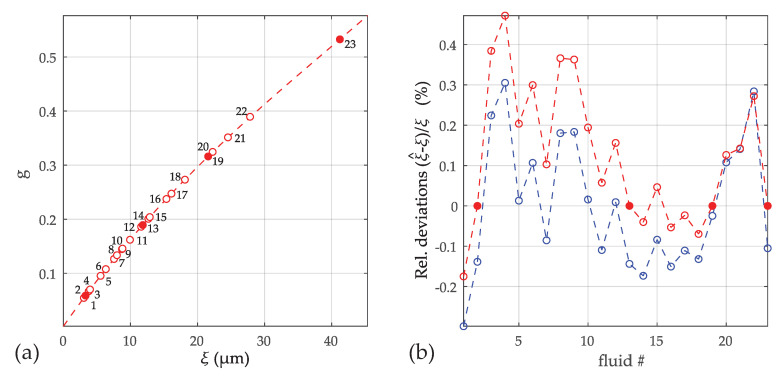
(**a**) Function *g* in Equation (24) for the liquids in Table 1. The fluids marked by the filled dots are used for estimating the constants $c_{1}\cdots c_{3}$ of a third order polynomial. (**b**) deviations between reference (ξ) and estimated values $\hat{\xi }$. These curves result if only the selected (red) or all fluids (blue) are used for calibrating the four model constants.

**Figure 8:**
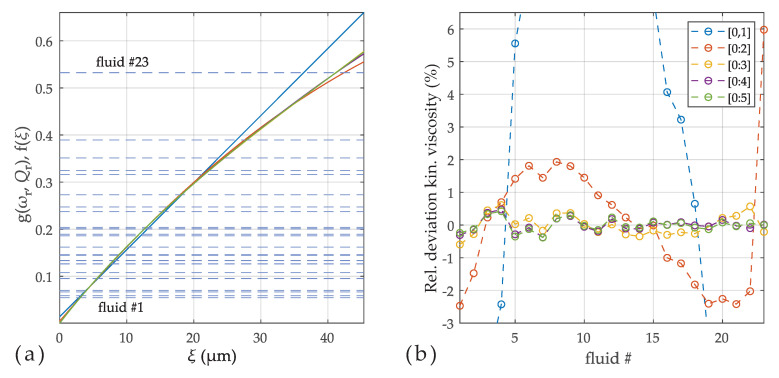
(**a**) The solutions $\xi^{*}$ are determined by the intersections of $g(\omega_{r},Q_{r})$ (dashed lines) and f(ξ) (solid lines). f(ξ) is shown for different polynomial orders, where all fluids were used for calibration. (**b**) The relative deviations between the reference (ν) and estimated ($\hat{\nu }$) kinematic viscosities decrease with increased order, but do not improve noticeably above an order of 4. The cut-off extreme deviations for first order are +10% and −22%.

The function *g* is calculated from the resonance parameters in Table 1 by

(29) $$\begin{array}{c} g\left(\omega_{r},Q_{r}\right)={\left(\frac{\omega_{0}}{\omega_{r}}-\frac{\omega_{r}}{\omega_{0}}\right)}^{-1}\left(\frac{\omega_{0}}{\omega_{r}}\frac{1}{Q_{r}}-\frac{1}{Q_{0}}\right) \end{array}$$

and is plotted over ξ in the left Figure 7. The fact that all measured fluids lie closely on a smooth line confirms the suitability of this model. The filled dots in Figure 7a indicate the fluids selected for model calibration. In (b), the deviations between calculated $\hat{\xi }$ and that of the reference values from Table 1 are shown for a third order polynomial. The deviations vanish therefore for these selected fluids. The blue results represent the deviations when all fluids were used to calibrate the four parameters of the polynomial. It can be observed that the agreement is not improved drastically, which is an indicator that these deviations are not model errors, but measurement errors and alterations of the test fluids from the certificate values. Figure 8 shows the consequence of reducing the order. The dashed lines on the left are the measured values *g* and the solid lines are the models for polynomial orders ranging from 1 to 5. (All fluids were used for these parameter fits.) The intersections of dashed and solid lines represent $\xi^{*}$. Although the number of roots of the used polynomials is equal to the order of the polynomial, it is observed that the intersections are unique, i.e., there is only one real root in the considered range, also for higher orders. In Figure 8b, the deviations between reference and estimated values of the kinematic viscosity are shown. Orders higher than four do not lead to further improvements in this case.

#### 3.2.3. Measurement of the Hydrodynamic Function of the QTF

**Figure 9:**
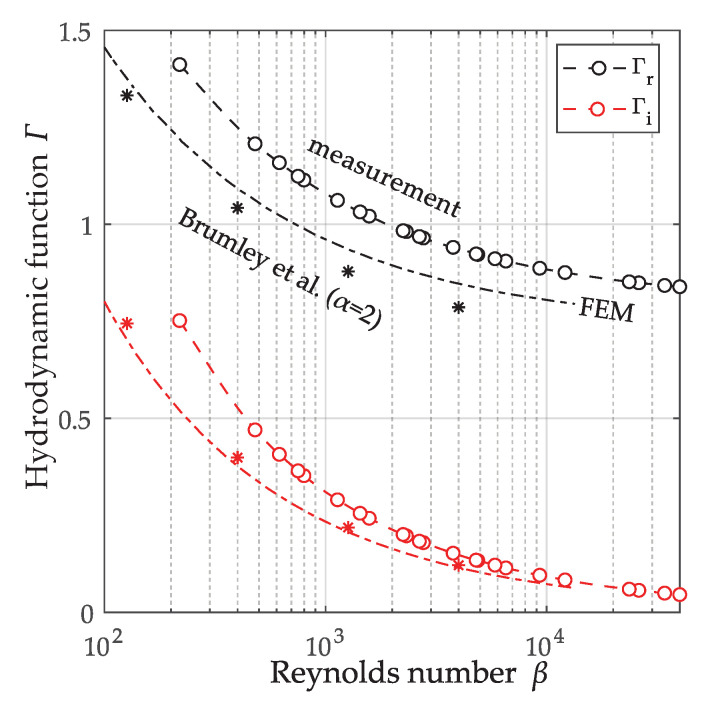
Estimated hydrodynamic function using Equation (A17) (${}^{\circ }$) and comparison with 2D simulation of rectangular cross-section (-·-) and tabulated values from [21] for aspect ratio α=2, which is the closet match to the actual α=1.74.

As was mentioned in Section 2, the approximation to the hydrodynamic function may be any suitable function and not necessarily a polynomial as used in this work. If for a given resonator a sufficient amount of measurements in various test fluids is available, the shape of the sampled hydrodynamic function can be obtained from resonance parameters by evaluating

(30) $$\underline{\Gamma }\left(\beta \right)=\frac{\rho_{s}}{\rho }\frac{\omega_{0}^{2}}{\omega_{r}^{2}}\left(1-\frac{\omega_{r}^{2}}{\omega_{0}^{2}}+j\frac{1}{Q_{0}}\frac{\omega_{r}}{\omega_{0}}-j\frac{1}{Q_{r}}\right),$$

i.e., Equation (A17) derived in Appendix B. The results are plotted in Figure 9 versus the Reynolds number β defined as

(31) $$\beta =\max{\left(W,H\right)}^{2}\omega \rho /\eta .$$

The density of the quartz material is $\rho_{s}=2649\;$kg/m${}^{3}$ and the width *W* and thickness *H* of one prong of the QTF are 610 μm and 350 μm, respectively (i.e., α=1.74). The result is compared the a 2D finite element simulation which was performed for a cantilever with the same cross-section using the solid mechanics module of COMSOL 5.3a ( The fluid properties were implemented by equivalent complex elastic moduli [33,38].). The (∗) represent the tabulated values from Brumley et al. [21] for an aspect ratio of α=2, where for the alternative definitions of the Reynolds number and the hydrodynamic function is accounted for by $\underline{\Gamma }=\alpha \pi {\underline{\Gamma }}_{\mathrm{Brumley}}^{*}/4$ and $\beta =4\beta_{\mathrm{Brumley}}$, with ${}^{*}$ denoting the complex conjugate. The deviations between 2D FEM simulation of the cantilever and the measurement results for the QTF may be attributed to the interaction and finite length of the QTF prongs. Furthermore, the not considered contribution of the fluid interaction at the end faces of the QTF is particularly strong, as vibration amplitudes are largest at the ends [39]. In addition, the resonator model was considered purely mechanical, but the neglected electrical interaction also affects the resonance parameters [40]. It can therefore be concluded that in most cases using the theoretical hydrodynamic functions without model calibration will not allow satisfactory accuracies for the determination of the fluid properties.

## 4. Conclusions

Fluid models providing a higher degree of approximation of the hydrodynamic function than the conventionally used ones were derived and applied to measurement results obtained with a quartz tuning fork sensor setup. These models demonstrate a clear advantage in terms of reduced model deviations for this particular problem, but it is assumed that they can be applied to many other types of vibrating sensors as well. Increased orders, however, do not significantly increase the complexity of the model calibration method and the viscosity and density calculation from measured resonance parameters.

## Figures and Tables

**Table 1:** Table of standard fluids in ascending order of ξ and the associated measured resonance parameters. The fluids used for model calibration are marked by ∗.

Fluid#	Fluid	Temp.	ξ	Density	Dyn. visc.	Kin. visc.	Res. freq.	*Q*-Factor
		(${}^{\circ }$C)	(μm)	(kg/m${}^{3}$)	(mPas)	(cSt)	(kHz)	(1)
1	N2	50	3.0508	740.1	1.2760	1.7241	29.482	95.869
2	* N2	40	3.3007	747.2	1.5060	2.0155	29.444	88.026
3	N2	25	3.7744	757.8	1.9930	2.6300	29.382	76.108
4	N2	20	3.9685	761.4	2.2120	2.9052	29.359	72.136
5	N7	50	5.5440	781.1	4.4050	5.6395	29.202	50.891
6	N7	40	6.3242	787.7	5.7680	7.3226	29.139	44.306
7	N14	50	7.5440	793.3	8.2420	10.390	29.054	36.975
8	N7	25	7.9856	797.5	9.2720	11.626	29.017	34.704
9	N7	20	8.7309	800.8	11.110	13.874	28.967	31.616
10	N14	40	8.8074	799.7	11.290	14.118	28.966	31.412
11	N26	50	9.9273	801.9	14.350	17.895	28.899	27.791
12	N14	25	11.592	809.3	19.670	24.305	28.788	23.563
13	* N26	40	11.835	808.2	20.470	25.328	28.779	23.120
14	N44	50	12.662	808.7	23.410	28.948	28.734	21.571
15	N14	20	12.881	812.5	24.320	29.932	28.711	21.118
16	N44	40	15.366	814.8	34.540	42.391	28.575	17.597
17	N26	25	16.114	817.6	38.050	46.539	28.527	16.715
18	N26	20	18.125	820.8	48.140	58.650	28.414	14.773
19	* N44	25	21.588	824.1	68.120	82.660	28.228	12.280
20	N140	50	22.259	819.1	71.950	87.840	28.218	11.938
21	N44	20	24.541	827.2	87.860	106.214	28.069	10.701
22	N140	40	27.840	825.1	112.20	135.984	27.923	9.3877
23	* N140	25	41.238	834.1	242.90	291.212	27.254	6.0978

## Appendix A. Dimensional Analysis

Dimensional analysis allows insights into the nature of a problem solely on basis of the involved variables and their physical units [41]. For this, all involved physical properties are collected and their scaling with length, time, and mass are noted.

(A1) $$\left[R\right]=L,\;\;\;\left[\omega \right]=T^{-1},\;\;\;\left[\rho \right]=L^{-3}\cdot M,\;\;\;\left[\eta \right]=L^{-1}\cdot T^{-1}\cdot M,\;\;\;\left[Z^{\prime }\right]=L^{-1}\cdot T^{-1}\cdot M.$$

The square bracket notation, e.g., [R]=L reads as “the dimension of *R* is *L*”. In this case *L* represents a dimensionless scaling factor that accounts for a change of units e.g., from meters to inch. By taking the logarithm of the dimensions of the physical quantities, a matrix H can be established

(A2) $$\left[\begin{array}{c} \log([R])\\ \log([\omega ])\\ \log([\rho ])\\ \log([\eta ])\\ \log(\left[Z^{\prime }\right]) \end{array}\right]=\underset{H}{\underset{︸}{\left[\begin{array}{ccc} 1 & 0 & 0\\ 0 & -1 & 0\\ -3 & 0 & 1\\ -1 & -1 & 1\\ -1 & -1 & 1 \end{array}\right]}}\cdot \left[\begin{array}{c} \log(L)\\ \log(T)\\ \log(M) \end{array}\right].$$

According to the Buckingham Π theorem [41], there are two dimensionless numbers in our case, forming the so-called π-group. The dimension of two is a consequence of having five parameters involved (the dependent variables) and only three basic physical scales (length, time and mass), which represent the independent variables. One, but not unique, representation of the π-group is obtained by calculating the kernel (null space) of the transpose of H

(A3) $$\ker\left(H^{T}\right)=\left[\begin{array}{cc} -2 & 2\\ -1 & 1\\ -1 & 1\\ 0 & -1\\ 1 & 0 \end{array}\right].$$

The number of dimensionless variables is therefore equal to the nullity of $H^{T}$. Each column vector contains the exponents of the dependent variables, such that their product is dimensionless, i.e.,

(A4) $$\begin{array}{c} \pi_{1}=\frac{Z^{\prime }}{R^{2}\omega \rho },\;\;\;\pi_{2}=\frac{R^{2}\omega \rho }{\eta }. \end{array}$$

As is outlined in [41], a function $f_{1}$ can be constructed such that

(A5) $$f_{1}\left(\pi_{1},\pi_{2}\right)=0,$$

which implies that one dimensionless variable is a function of the (all) other, i.e.,

(A6) $$\pi_{1}=f_{2}\left(\pi_{2}\right).$$

Solving Equation (A4) for $Z^{\prime }$ yields therefore

(A7) $$Z^{\prime }=R^{2}\omega \rho \;\pi_{1}\left(\pi 2\right).$$

By comparing this result with the solution in Equation (1), $\pi_{1}$ is identified as $j\pi \underline{\Gamma }$ and $\pi_{2}=\beta$ as the Reynolds number. Therefore, the basic structure of the fluid impedance could be revealed by dimensional analysis alone.

The idea is now applied to the more complex situation of a cantilever of rectangular cross-section mounted in a tube of radius *R* as shown in Figure 1. Performing a dimensional analysis with these parameters adds two aspect ratios as dimensionless variables e.g.,

(A8) $$\pi_{3}=W/H=\alpha_{1},\;\;\;\mathrm{and}\;\;\;\pi_{4}=W/R=\alpha_{2}.$$

In this case, the fluid impedance per length, is

(A9) $${\underline{Z}}^{\prime }=j\omega A\rho \;\underline{\Gamma }\left(\beta ,\alpha_{1},\alpha_{2}\right).$$

Consequently, the hydrodynamic function $\underline{\Gamma }$ is, no matter how complex the geometry actually is, always a function of the Reynolds number β and all the aspect ratios which appear in the considered problem. For fluid sensing applications, the geometry of the sensor is fixed, but the fluid parameters ρ and ν appearing in the fore factor and in the hydrodynamic function are variable. Therefore, for any vibrating cross-section and configuration the form

(A10) $${\underline{Z}}^{\prime }=j\omega A\rho \;\underline{\Gamma }\left(\xi \right),$$

can be expected, where *A* is a parameter of dimension $L^{2}$ which is chosen to coincide with the area of the vibrating cross-section, as it will turn out to be convenient in Appendix B. The fluid dependent factor in the Reynolds number β defines a new variable $\xi =\sqrt{\nu /\omega }$, which is used henceforth.

It is mentioned for the sake of completeness that properties which are associated with the finite compressibility of the fluid, such as the bulk modulus and the longitudinal viscosity [42] were not considered, but would extend the π-group. Fluid sensors for density and viscosity sensing, are typically designed such that these influences need not to be considered.

## Appendix B. Resonance Parameters

The deflection amplitude $\underline{u}(x,\omega )$ of a single or double clamped prismatic beam of length *L* can be expressed by superposition of modeshapes $\phi_{i}\left(x\right)$ times generalized coordinates ${\underline{q}}_{i}\left(\omega \right)$

(A11) $$\underline{u}\left(x,\omega \right)=\sum_{i=0}^{\infty }\phi \left(x\right){\underline{q}}_{i}\left(\omega \right).$$

The equivalent mass and the generalized force due to fluid loading for one particular mode of vibration can be determined from considering energy balances, yielding

(A12) $$\begin{array}{cc} {\bar{m}}_{i} & =\rho_{s}A\int_{0}^{L}\phi_{i}{\left(x\right)}^{2}dx, \end{array}$$

(A13) $$\begin{array}{cc} {\underline{F}}_{i}\left(\omega \right) & =\int_{0}^{L}\phi \left(x\right){\underline{F}}_{i}^{\prime }\left(x,\omega \right)dx=-\omega^{2}{\underline{q}}_{i}\left(\omega \right)\rho A\underline{\Gamma }\;\int_{0}^{L}\phi_{i}{\left(x\right)}^{2}dx=-\omega^{2}\frac{\rho }{\rho_{s}}m_{i}\underline{\Gamma }{\underline{q}}_{i}\left(\omega \right). \end{array}$$

When the area *A* in the fore factor in Equation (A9) is chosen to coincide with the area of the resonator cross-section, they cancel out. Splitting the complex hydrodynamic function in $\underline{\Gamma }=\Gamma_{R}-j\Gamma_{I}$ and adding the fluid force to the spring-mass damper system driven with the generalized force ${\underline{F}}_{0}$, yields

(A14) $$\begin{array}{cc} {\underline{F}}_{0} & =\left(-\omega^{2}m_{i}+j\omega c_{i}+k_{i}\right){\underline{q}}_{i}\left(\omega \right)+{\underline{F}}_{i} \end{array}$$

(A15) $$\begin{array}{cc} \; & =\left(-\omega^{2}m_{i}\left(1+\frac{\rho }{\rho_{s}}\Gamma_{R}\right)+j\omega \left({\bar{c}}_{i}+\omega \frac{\rho }{\rho_{s}}m_{i}\Gamma_{I}\right)+k_{i}\right){\underline{q}}_{i}\left(\omega \right). \end{array}$$

As a consequence, the resonance parameters of the fluid loaded resonator can be given solely in terms of vacuum resonance parameters $\omega_{0}$ and $Q_{0}$ and the hydrodynamic function scaled with the ratio of fluid density ρ to resonator density $\rho_{s}$

(A16) $$\begin{array}{c} \omega_{r}=\frac{\omega_{0}}{\sqrt{1+\frac{\rho }{\rho_{s}}\Gamma_{R}}}\;\;\;\mathrm{and}\;\;\;Q_{r}=\frac{\omega_{0}}{\omega_{r}}{\left(\frac{1}{Q_{0}}+\frac{\omega_{r}}{\omega_{0}}\frac{\rho }{\rho_{s}}\Gamma_{I}\right)}^{-1}. \end{array}$$

Given Equation (A16), the hydrodynamic function can be determined from measurements in known test liquids by

(A17) $$\underline{\Gamma }\left(\beta \right)=\frac{\rho_{s}}{\rho }\frac{\omega_{0}^{2}}{\omega_{r}^{2}}\left(1-\frac{\omega_{r}^{2}}{\omega_{0}^{2}}+j\frac{1}{Q_{0}}\frac{\omega_{r}}{\omega_{0}}-j\frac{1}{Q_{r}}\right).$$

For unknown sensor parameters, the scaling of $\underline{\Gamma }$ and β remain unknown, but the complexity of $\underline{\Gamma }$ and the required order of approximation can readily be assessed from measurements. These scaling factors are implicitly accounted for by the model parameter calibration procedures in Section 2.4 and Section 2.5.
